# Enzymatic Hydrolysis of Alginate to Produce Oligosaccharides by a New Purified Endo-Type Alginate Lyase

**DOI:** 10.3390/md14060108

**Published:** 2016-06-06

**Authors:** Benwei Zhu, Meijuan Chen, Heng Yin, Yuguang Du, Limin Ning

**Affiliations:** 1 Preclinical Medicine College, Nanjing University of Chinese Medicine, Nanjing 210023, China; zhubenwei@njtech.edu.cn (B.Z.); meijuanchen@126.com (M.C.); 2 College of Food Science and Light Industry, Nanjing University of Technology, Nanjing 211816, China; 3 Dalian Institute of Chemical Physics, Chinese Academy of Sciences, Dalian 116023, China; yinheng@dicp.ac.cn; 4 Institute of Process Engineering, Chinese Academy of Sciences, Beijing 100190, China; ygdu@ipe.ac.cn

**Keywords:** alginate lyase, *Cellulophaga*, enzymatic hydrolysis, oligosaccharides

## Abstract

Enzymatic hydrolysis of sodium alginate to produce alginate oligosaccharides has drawn increasing attention due to its advantages of containing a wild reaction condition, excellent gel properties and specific products easy for purification. However, the efficient commercial enzyme tools are rarely available. A new alginate lyase with high activity (24,038 U/mg) has been purified from a newly isolated marine strain, *Cellulophaga* sp. NJ-1. The enzyme was most active at 50 °C and pH 8.0 and maintained stability at a broad pH range (6.0–10.0) and temperature below 40 °C. It had broad substrate specificity toward sodium alginate, heteropolymeric MG blocks (polyMG), homopolymeric M blocks (polyM) and homopolymeric G blocks (polyG), and possessed higher affinity toward polyG (15.63 mM) as well as polyMG (23.90 mM) than polyM (53.61 mM) and sodium alginate (27.21 mM). The TLC and MS spectroscopy analysis of degradation products suggested that it completely hydrolyzed sodium alginate into oligosaccharides of low degrees of polymerization (DPs). The excellent properties would make it a promising tool for full use of sodium alginate to produce oligosaccharides.

## 1. Introduction

Alginate is a linear acidic polysaccharide consisting of 1,4-linked β-d-mannuronate (M) and its C5 epimer α-l-guluronate (G) [1]. These uronate residues are arranged as homopolymeric M blocks (polyM) and homopolymeric G blocks (polyG), and heteropolymeric MG blocks (polyMG) [1]. Approximately 30,000 tons of alginate is produced annually, which is estimated to be less than 10% of the total amount of biosynthesized alginate [2]. Hence, alginate may be an abundant resource of biomaterials in the future. Alginate is an important source for food and medicine. It possesses various kinds of physiological activities, such as anti-tumor [3,4], immune-regulating [5,6] and growth-promoting [7]. It is also involved in the repair of injury of skin [8] as well as immobilization of enzymes and cells [9]. However, the application of alginate in medicine has been greatly limited by its gelation. Alginate oligosaccharides, the hydrolysates of alginate, have now attracted increasing attention due to their obvious activities, excellent gel properties and good solubility, which is essential for absorption. They can be used as growth promoters for plants and therapeutic agents such as anticoagulants and tumor inhibitors [10]. They can also induce the production of cytokines and regulate the blood sugar as well as lipids [11]. As a result, the preparation of alginate oligosaccharides by enzymatic hydrolysis has been the focus of various fields.

Alginate lyase can degrade alginate by β-elimination of glycosidic bonds and produce unsaturated oligosaccharides with double bonds at the non-reducing end [12]. A number of alginate lyases have been identified, had their genes cloned, and were purified and characterized from various sources, including marine and terrestrial bacteria, marine mollusks and algae. They can be classed into two groups due to their substrate specificities [12]: one is the G block–specific lyase (polyG lyase, EC4.2.2.11), and the other is the M block–specific lyase (polyM lyase, EC4.2.2.3). This classification has been widely accepted, but some enzymes show activities toward both polyM and polyG [13,14,15,16,17,18], which may degrade alginate more effectively. In terms of the mode of action, alginate lyase can be grouped into endolytic and exolytic alginate lyase [12]. Endolytic alginate lyase cleaves glycosidic bonds inside alginate polymer and releases unsaturated oligosaccharides (di-, tri-, and tetra-saccharides) as the main products [19], while exolytic alginate lyase can further degrade oligosaccharides into monomers from the non-reducing end [20,21,22]. Now alginate lyases, especially endolytic alginates, have been widely used in the production of alginate oligosaccharides [23], the elucidation of the fine structures of alginate [24] and the preparation of protoplasts of red and brown algae [25,26]. Furthermore, alginate lyase also shows great potential application in the treatment of cystic fibrosis by degrading the polysaccharide biofilm of bacterium [27]. Therefore, considering the lack of commercial enzymes with excellent characteristics, it is of great urgency to obtain alginate lyase with high activity and broad substrate specificity.

In this work, a new alginate lyase with broad substrate specificity has been purified and characterized from the marine bacteria *Cellulophaga* sp. NJ-1. The kinetics and analysis of degrading products were also characterized, suggesting that it would be a potential candidate for expanding the application of alginate lyases.

## 2. Results and Discussion

### 2.1. Isolation of Alginate-Degrading Strain

**Figure 1:**
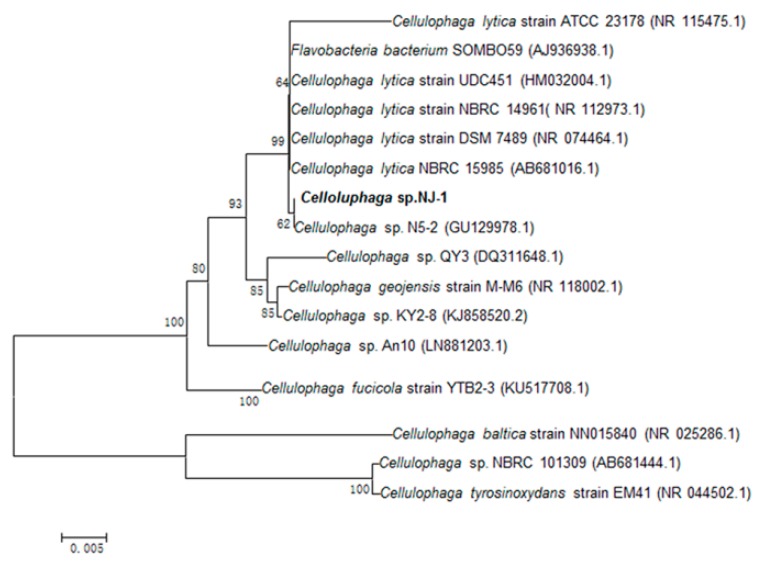
The phylogenetic tree of strain NJ-1 and related bacteria based on a maximum parsimony analysis of 16S rDNA sequences.

The strain was from rotten red algae from the Yellow Sea. The 16S rDNA sequence of the strain was sequenced and submitted to GeneBank (accession number KU168595). According to the phylogenetic analysis of 16S rDNA sequence, the strain was assigned to the genus *Cellulophaga* and named *Cellulophaga* sp. NJ-1 (Figure 1).

### 2.2. Purification of Alginate Lyase

**Figure 2:**
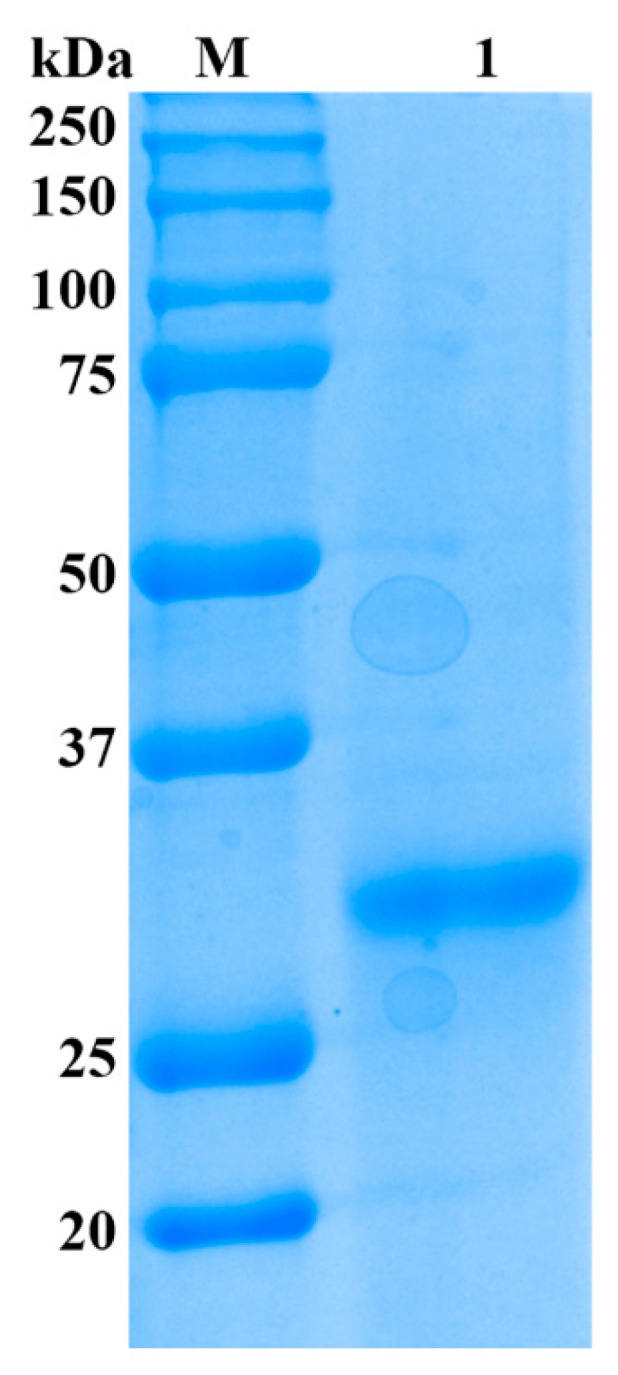
SDS-PAGE analysis of purified enzyme from *Cellulophaga* sp. NJ-01. Lane M, protein ruler; Lane 1, purified alginate lyase.

As shown in Table 1, the alginate lyase was purified by a series of purification procedures and finally yielded significantly high activity of 24,178 U/mg towards sodium alginate, which resulted in a 318.1-fold purification, while only 6.7% recovery was achieved. The result of SDS-PAGE showed that the enzyme was purified as a single protein band with a molecular mass of 32 kDa (Figure 2) and designated as Cel32. The alginate lyase AlySJ-02 from *Pseudoalteromonas* sp. SM0524 [15], the alginate lyase from *Pseudoalteromonas* sp. NO272 [16] and the A1m from *Agarivorans* sp. JAM-A1m [28] have similar molecular weights of 32 kDa, 33.9 kDa and 31 kDa, respectively. The different enzymes from different microorganisms differ in size from 23 kDa to 110 kDa. Those alginate lyases can be grouped into three classifications based on their molecular masses: small (25–30 kDa), medium-sized (around 40 kDa) and large lyases (>60 kDa) [15]. The enzyme in this study belonged to the small alginate lyase.

### 2.3. Substrate Specifities and Enzymatic Kinetics of the Enzyme

Four kinds of substrates were used to investigate the substrate specificity of the enzyme (Table 2). The alginate lyase showed higher activity toward sodium alginate and polyM than to polyG and polyMG. The enzyme showed high activities toward the four kinds of substrates. The results above indicated that it possessed broader substrate specificity compared with other alginate lyases and was a new member of the bifunctional alginate lyases.

The kinetic parameters of alginate lyase toward sodium alginate, polyMG, polyM and polyG were estimated from a series of steady-state initial reaction rates *V*_0_ (pmol/s) measured at various substrate concentrations. As shown in Table 2, the *K*_m_ values of Cel32 with sodium alginate, polyMG, polyM and polyG as substrates were 27.21 mM, 23.90 mM, 53.61 mM and 15.62 mM, respectively. The *K*_cat_ values of Cel32 with sodium alginate, polyMG, polyM and polyG as substrates were 101.87 s^−1^, 81.64 s^−1^, 163.73 s^−1^, and 51.78 s^−1^. It had much lower *K*_m_ values towards polyG and polyMG, indicating that it showed higher affinity toward the G block of the substrates.

### 2.4. Biochemical Properties of the Enzyme

**Figure 3:**
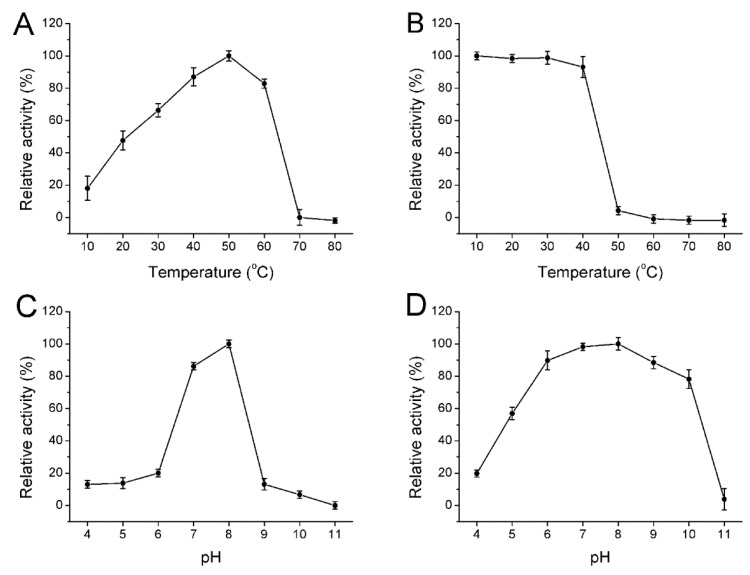
Biochemical characterization of the enzyme. (**A**) The optimal temperature of the enzyme; (**B**) Thermal stability of Cel32; (**C**) The optimal pH of Cel32; (**D**) The pH stability of Cel32. The highest activity was set to be 100%. Each value represents the mean of three replicates ± standard deviation.

The biochemical characterization of the enzyme was further identified. The enzyme showed maximum activity at 50 °C (Figure 3A) and was stable below 40 °C (Figure 3B). This enzyme possessed approximately 90% activity after incubation at 40 °C for 30 min and was gradually inactivated as temperature increased. The optimal temperatures for alginate lyase from *Pseudoalteromonas* sp. NO272 [16] and A1m from *Agarivorans* sp. JAM-A1m [28] were only 25 °C and 30 °C, respectively, while other enzymes such as AlySJ-02 from the *Pseudoalteromonas* sp. SM0524 [15] and alginate lyases from *Isoptericola halotolerans* CGMCC 5336 [14] have a higher optimal temperature of 50 °C. The optimal pH for the enzyme activity was 8.0 (Figure 3C) and retained more than 80% activity at a broad pH range from pH 6.0 to 10.0 (Figure 3D) after incubation for 24 h. However, this enzyme was mostly stable at pH 8.0 and retained more than 80% activity at a broad pH range of pH 6.0 to 10.0. Thus, Cel32 was an alkaline-stable lyase and it could retain stability in a broader pH range.

**Figure 4:**
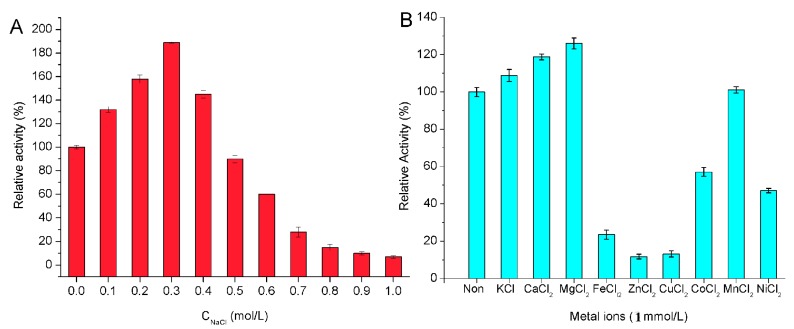
The effect of NaCl (**A**) and metal ions (**B**) on the activity of the enzyme.

The effect of NaCl on the activity of Cel32 towards sodium alginate was shown in Figure 4A. The activity of Cel32 was greatly enhanced by NaCl, which indicated that the enzyme is a salt-activated alginate lyase. Interestingly, AlyYKW-34 from *Vibrio* sp. YKW-34 is a Na^+^/K^+^-dependent lyase but its activity cannot be further affected by the concentration of these two metal ions [29]. Therefore, alginate lyases from different species may be affected by different ions due to the different environment where the bacteria strains survived and evolved.

The effects of metal ions (1 mM) on the enzyme were performed in buffers without NaCl. As shown in Figure 4B, Ca^2+^, Mg^2+^ and K^+^ showed an activation effect on the enzyme activity. Among the divalent metal ions, Mg^2+^ displayed the most stimulating effect with 128% of relative activity followed by Ca^2+^ with 119%, while the metal ions such as Fe^2+^, Cu^2+^, Zn^2+^, Co^2+^, and Ni^2+^ showed inactivation effects on enzyme activity. Other metal ions investigated, such as Mn^2+^, displayed a slight effect on the enzyme activity. The effects of metal ions on Cel32 were similar to that on FlAlyA from *Flavobacterium* sp. Strain UMI-01 [30] and alginate lyase from *Isoptericola halotolerans*. CGMCC5336 [14]. It has been reported that Ca^2+^ and Mg^2+^ could protect the enzyme against thermal denaturation and play a vital role in maintaining the active conformation of the enzyme at high temperatures [31]. As for other divalent metal ions, they chelated with the residues of catalytic sites and then covered the active center.

### 2.5. The Action Modes and Substrate Binding Subsites of the Enzyme

**Figure 5:**
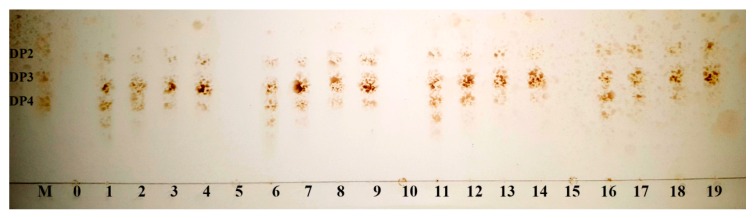
TLC analysis of degradation products of the enzyme toward sodium alginate, polyMG, polyM and polyG. Lane M: purified oligosaccharides (dimers, trimers, and tetramers); Lane 0–4: hydrolysates of sodium alginate for 0, 3, 12, 24 and 36 h; Lane 5–9: hydrolysates of polyMG for 0, 3, 12, 24 and 36 h; Lane 10–14: hydrolysates of polyM for 0, 3, 12, 24 and 36 h; Lane 15–19: hydrolysates of polyG for 0, 3, 12, 24 and 36 h.

The degradation products of the four kinds of substrates by Cel32 were analyzed by TLC plate (Figure 5). As the proceeding of hydrolysis, oligosaccharides with low DP (2–6) appeared. After incubation for 36 h, the dimers and trimers were the main hydrolysis products for sodium alginate, polyMG, polyM and polyG. The distributions of the degradation products for the above four kinds of substrates were similar, and results indicated that Cel32 hydrolyzes the substrates in an endolytic manner.

To determine the number of substrate binding subsites in the active tunnel of Cel32, we compared the degrading capability of Cel32 on oligosaccharide substrates with different DPs (data not shown). The purified disaccharides and trisaccharides were not further degraded by Cel32 even under more focused conditions (high enzyme concentration and prolonged incubation time). The tetrasaccharide was the shortest chain that can be recognized and cleaved by Cel32, producing disaccharide only. However, the tetrasaccharide and pentasaccharide were not degraded completely by Cel32 compared with oligosaccharides with DP 6–8, and the result indicated that hexasaccharide was the optimal substrate for Cel32.

### 2.6. Enzymatic Hydrolysis of Sodiumalginate by Using Cel32

**Figure 6:**
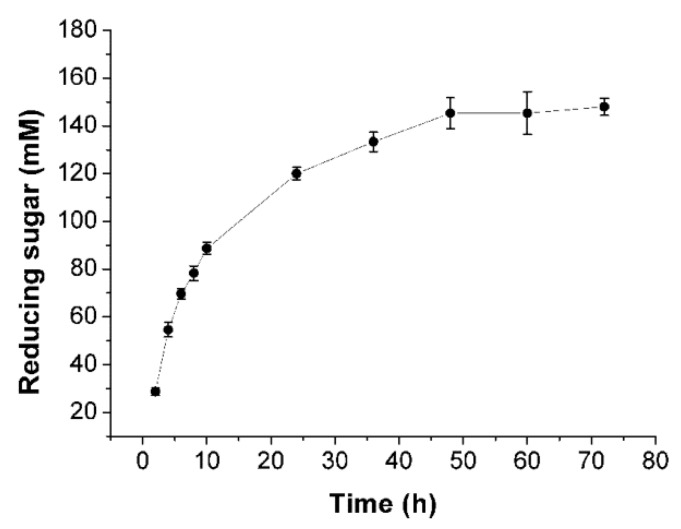
Enzymatic hydrolysis of sodium alginate by Cel32. The course of hydrolytic procedure was monitored by determining the amount of reducing sugars during the hydrolysis.

In order to investigate the hydrolytic ability of Cel32, the course of the hydrolytic procedure was monitored by determining the amount of reducing sugars during the hydrolysis. As shown in Figure 6, at the initial stage of hydrolysis (2–24 h), the amount of reducing sugars increased dramatically due to the enzyme breakdown of the sodium alginate endolytically to release a large amount of oligosaccharides. In contrast, after hydrolysis for 36 h, there was no obvious increase in the yield of reducing sugars, partially because the enzyme lost its activity after being incubated at 30 °C for a long time.

### 2.7. ESI-MS Analysis of the Degradation Products of Cel32

**Figure 7:**
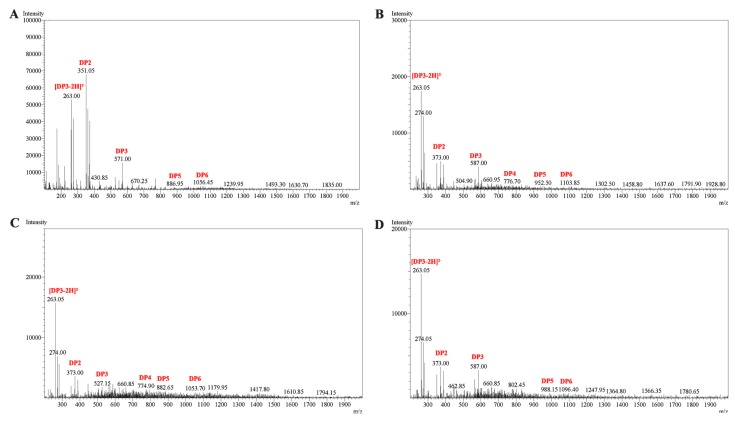
ESI-MS analysis of the Cel32 hydrolysis products with (**A**) sodium alginate; (**B**) polyMG; (**C**) polyM; and (**D**) polyG as substrates. DP indicates the degree of depolymerization of alginate oligosaccharides released from the substrates.

In order to further determine the composition of the degradation products, the hydrolysates were then loaded onto a carbograph column to remove salts after removing other proteins, followed by being concentrated, dried and redissolved in 1 mL methanol. The degradation products were then analyzed by ESI-MS. As shown in Figure 7, disaccharides and trisaccharides account for a major fraction of the hydrolysates of four kinds of substrates. The product distribution of AlySJ-02 [15] was similar to Cel32, while other bifunctional enzymes mainly produced oligosaccharides with DPs of 2–5 during the hydrolysis of the sodium alginate [14]. The commercial enzyme originating from *Flavobacterium* sp. preferred polyG over polyM and degraded sodium alginate into penta- to heptasaccharides [32]. This result indicated that Cel32 may be a potential tool for the enzymatic hydrolysis of sodium alginate to produce oligosaccharides with lower DPs.

## 3. Materials and Methods

### 3.1. Microorganism

The alginate-degrading bacterial strains used in this study were isolated from rotten seaweed of Yellow Sea.

### 3.2. Media and Culture Conditions

All chemicals were of regent grade. Sodium alginate (*Macrocystis* *pyrifera* origin, *M*/*G* ratio 77/23) was purchased from Sigma-Aldrich (St. Louis, MO, USA). PolyM (*M*/*G* ratio 97.3/2.7, purity: about 99%), polyG (*M*/*G* ratio 1.8/98.2, purity: about 99%) and oligosaccharides with DPs from 2 to 8 were purchased from Qingdao BZ Oligo Biotech Co., Ltd. (Qingdao, China). PolyMG (*M*/*G* ratio 37/63) was donated by Doctor Dongsheng Sun of Dalian Institute of Chemical Physics, Chinese Academy of Sciences. Sodium alginate minimal medium containing 5.0 g/L sodium alginate, 5.0 g/L (NH_4_)_2_SO_4_, 2.0 g/L K_2_HPO_4_, 30.0 g/L NaCl, 1.0g/L MgSO_4_·7H_2_O, 0.01 g/L FeSO_4_·7H_2_O at pH 7.0 for stain isolation. An optimized fermentation medium for growth enrichment was composed of 6.0 g/L sodium alginate, 5.0 g/L tryptone, 2.5 g/L yeast extract, 25.0 g/L NaCl, 2 mM MgSO_4_, 0.5 mM CaCl_2_, 1 mM KH_2_PO_4_, 0.2 mM FeSO_4_, and 0.3 mM MnSO_4_ at pH 8.0. Solid medium was prepared by adding 20 g/L of agar to the above medium.

### 3.3. Strain Isolation and Identification

The samples were collected from the coast of the Yellow Sea, washed by sterilized sea water and then spread on sodium alginate-agar plates. The plates were incubated at 30 °C for 36 h and the positive colonies showing clear zones were picked out from the selection plates. The re-screening process was conducted as follows. Strains with clear hydrolytic zones were selected and incubated aerobically in a fermentation medium at 30 °C and 200 rpm. Furthermore, the activity of alginate lyase was determined by 3,5-dinitrosalicylic acid (DNS) colorimetry [33]. Among the isolates, the most active strain NJ-1 was selected for further studies.

The 16S rDNA sequence of the stain was amplified by PCR from the genomic DNA and then sequenced. The forward primer was designed as 5′-AGAGTT TGATCCTGGCTCAG-3′ and the reverse primer was designed as 5′-TACGGCTACCTTGTTACGACTT-3′. The sequence was blasted and aligned with closely related sequences retrieved from NCBI using the BLASTn and CLUSTAL X program, respectively.

### 3.4. Enzyme Purification

The strain was propagated in fermentation medium with shaking for 48 h at 30 °C. The culture medium was centrifuged (10,000× *g*, 60 min) and the cell-free supernatant was fractionated at 40% and 80% ammonium sulfate saturation. The precipitated protein with 40% ammonium sulfate saturation was discarded, and the precipitated protein with 80% ammonium sulfate saturation was suspended in distilled water and dialyzed in dialysis bag (MWCO: 8000–14,000 Da) against the distilled water, and freeze-dried successively. Protein contents were determined by Bradford method [34]. The obtained enzyme powder was dissolved in 5 mL Tris-HCl buffer (pH 8.0) with 4% as the final concentration, then the enzyme solution was applied to Sephadex G-200 (Pharmacia Company, Stockholm, Sweden) column chromatography (100 × 1.6 cm) equilibrated with the same buffer, and eluted at a flow rate of 0.1 mL/min. The eluents were monitored continuously at 280 nm for protein and fractions were assay for activity against sodium alginate. All the fractions of the first peak from Sephadex G-200 column chromatography containing alginate lyase activity were gathered, concentrated and applied to another column of Sephadex G-75, furthermore equilibrated with the same eluent. Fractions were collected and monitored for the presence of alginate lyase. The purity of the fractions was assessed by SDS-PAGE. Pure fractions with activity were stored at −20 °C.

### 3.5. Assay for Alginate Lyase Activity and Protein Concentration

Purified enzyme (0.1 mL) was mixed with 0.9 mL Tris-HCl (20 mM, pH 8.0, 1% sodium alginate) and incubated at 30 °C for 10 min. The reaction was stopped by heating in boiling water for 10 min. The enzyme activity was then assayed by measuring the increased absorbance at 235 nm due to the formation of double bond between C4 and C5 at the non-reducing terminus by β-elimination. One unit was defined as the amount of enzyme required to increase the absorbance at 235 nm by 0.01 per min.

### 3.6. Substrate Specificity and Kinetic Measurement of Alginate Lyase

The purified enzyme was reacted with 1% of sodium alginate, polyMG, polyM and polyG, respectively. The assay of enzyme activity was defined as described previously.

The kinetic parameters of the purified enzyme toward these four kinds of substrates were determined by measuring the enzyme activity with substrates at different concentrations (0.1–10.0 mg/mL). The *K*_m_ and *V*_max_ values were calculated by using linear regression plots of Lineweaver and Burk [35].

### 3.7. Biochemical Characterization of the Enzyme

The effects of pH on the enzyme activity were evaluated by incubating the purified enzyme in buffers with different pH (4.0–11.0) under the assay conditions described previously. The pH stability depended on the residual activity after the enzyme was incubated in buffers with different pH (2.0–11.0) for 24 h. Meanwhile, the effects of temperature (20–80 °C) on purified enzyme were investigated at pH 8.0. The thermal stability of the enzyme was determined under the assay conditions described previously after incubating the purified enzyme at 20–80 °C for 30 min. The buffers with different pH were used phosphate-citrate (pH 2.0–5.0), Tris–HCl (pH 6.0–9.0) and Na_2_HPO_4_–NaOH (pH 10.0–11.0).

The effects of NaCl on enzyme activity were measured in buffers with different concentrations of NaCl (100–1000 mM) under standard test conditions. The enzyme activity without NaCl served as control. The influence of metal ions on the activity of the enzyme were performed by incubating the purified enzyme at 4 °C for 24 h in the presence of various metal compounds at a concentration of 1 mM. Then the activity was measured under standard test conditions. The reaction mixture without any metal ion was taken as control.

### 3.8. Analysis of Action Mode and Substrate Binding of the Enzyme

To determine the smallest substrate and the number of substrate binding subsites in its catalytic tunnel of alginate lyase, hydrolysis reactions were carried out using oligosaccharides with different DPs (DP 2 to 8) at a concentration of 10 mg/mL in 10 μL reaction mixture (pH 7.0). The reaction mixtures were incubated at 30 °C with enzyme for 12 h. After incubation, the mixture solutions were boiled for 10 min and then centrifuged at 12,000 rpm for 10 min to remove the unsolved materials. The hydrolysates were loaded onto a carbograph column (Alltech, Grace Davison Discovery Sciences, Carnforth, UK) to remove salts after removing proteins, and then concentrated, dried and re-dissolved in 1 mL methanol. The degradation products were firstly analyzed by TLC with the solvent system (1-butanol/formic acid/water 4:6:1) and visualized by heating TLC plate at 130 °C for 5 min after spraying with 10% (*v*/*v*) sulfuric acid in ethanol.

For investigating the action mode, the reaction mixtures (800 μL) containing 1 μg purified enzyme and 2 mg substrates (sodium alginate, polyMG, polyM or polyG) were incubated at 30 °C for 0–36 h. The hydrolysis products were analyzed by TLC as above.

### 3.9. Enzymatic Depolymerization of Sodium Alginate

About 1 mL of enzyme was mixed with 100 mL 1% sodium alginate supplemented with 0.01% (*w*/*v*) NaN_3_ and 40 μg/mL tetracycline, and then the mixture was incubated at 30 °C. The aliquot samples were taken at 2, 4, 6, 8, 10, 24, 36, 48, 60 and 72 h to determine the amount of reducing sugars by using the 3,5-dinitrosalicylic acid (DNS) colorimetry.

### 3.10. ESI-MS Analysis of the Degradation Products of the Enzyme

To further determine the composition and degree of polymerization (DP) of the products, ESI-MS was used. 2 μL supernatant was loop-injected to an LTQ XL linear ion trap mass spectrometer (Thermo Fisher Scientific, Waltham, MA, USA) after centrifugation. The oligosaccharides were detected in a negative-ion mode using the following settings: ion source voltage, 4.5 kV; capillary temperature, 275–300 °C; tube lens, 250 V; sheath gas, 30 arbitrary units (AU); scanning the mass range, 150–2000 *m*/*z*.

## 4. Conclusions

A new alginate lyase with high activity has been purified from *Cellulophaga* sp. NJ-1. It had broad substrate specificity toward sodium alginate, polyMG, polyM and polyG. The enzyme possessed higher activity towards polyM and sodium alginate than polyG and polyMG. The enzyme showed maximal activity at 50 °C and pH 8.0 and maintained stability at a broad pH range and temperature below 40 °C. The degradation products analysis suggested that it could completely hydrolyze sodium alginate into oligosaccharides of low DPs. Moreover, the excellent thermal stability and high activity would make it a potent tool to produce oligosaccharides with bioactivities.

## Figures and Tables

**Table 1:** Summary for purification of Cel32.

Step	Total Protein (mg)	Total Activity (U)	Specific Activity * (U/mg)	Folds	Recovery (%)
Crude enzyme	1331	100,548	76	1	100
(NH_4_)_2_SO_4_ precipitation	73.2	37,625	514	6.8	37.4
SephadexG-200-filtration	0.73	9577	13,042	172.6	9.5
SephadexG-75-filtration	0.28	6770	24,083	318.1	6.7

* Note: One unit was defined as the amount of enzyme required to increase the absorbance at 235 nm by 0.01 per min.

**Table 2:** Specific activities and kinetic parameters of Cel32 toward sodium alginate, polyMG, polyM and polyG.

Substrate	Sodium Alginate	PolyMG	PolyM	PolyG
Specific activity * (U/mg)	24,083.21	15,952.55	30,665.69	15,970.07
*K*_m_ (mM)	27.21	23.90	53.61	15.62
*V*_max_ (nmol/s)	4.37	3.50	7.02	2.22
*K*_cat_ (s^−1^)	101.87	81.64	163.73	51.78
*K*_cat_*/K*_m_ (s^−1^/mM)	3.74	3.42	3.05	3.31

* Note: One unit was defined as the amount of enzyme required to increase the absorbance at 235 nm by 0.01 per min.
